# Second harmonic generation and nonlinear frequency conversion in photonic time-crystals

**DOI:** 10.1038/s41377-025-01788-z

**Published:** 2025-04-02

**Authors:** Noa Konforty, Moshe-Ishay Cohen, Ohad Segal, Yonatan Plotnik, Vladimir M. Shalaev, Mordechai Segev

**Affiliations:** 1 Physics Department, Technion—Israel Institute of Technology, Haifa, Israel; 2 Solid State Institute, Technion—Israel Institute of Technology, Haifa, Israel; 3 Department of Electrical and Computer Engineering, Technion—Israel Institute of Technology, Haifa, Israel; 4 School of Electrical and Computer Engineering, Birck Nanotechnology Center and Purdue Quantum Science and Engineering Institute, Purdue University, West Lafayette, IN USA; 5 Quantum Science Center (QSC), National Quantum Information Science Research Center of the U.S. Department of Energy (DOE), Oak Ridge, TN USA

**Keywords:** Nonlinear optics, Nonlinear optics

## Abstract

We study the nonlinear process of second harmonic generation in photonic time-crystals, materials with refractive index that varies abruptly and periodically in time, and obtain the phase matching condition for this process. We find conditions for which the second harmonic generation is highly enhanced even in the absence of phase matching, governed by the exponential growth of the modes residing in the momentum gap of the photonic time crystal. Additionally, under these conditions, a cascade of higher-order harmonics is generated at growing exponential rates. The process is robust, with no requirement for phase-matching, the presence of a resonance or a threshold, drawing energy from the modulation.

## Abstract (web summary)

We study second harmonic generation in photonic time-crystals, and find conditions for which the process is highly enhanced, leading to efficient generation of higher-order harmonics.

## Graphical abstract

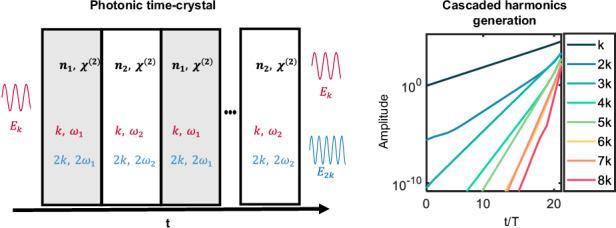

## Introduction

The exploration of epsilon-near-zero materials presents new opportunities to create time-interfaces with large and abrupt changes in the refractive index^1–11^. The recent advancements in theory and experiments have drawn increasing attention to photonic time-crystals (PTCs)^12–21^. PTCs are materials with a refractive index that undergoes substantial periodic variations on the time scales of a single optical cycle. They exhibit dispersion relation displaying momentum bands, separated by momentum gaps wherein the electromagnetic (EM) modes are exponentially growing (or decaying) in time^12,18–21^. The study of PTCs, and generally of time-varying media, is introducing new avenues for shaping light-matter interactions, relevant both for lasing technologies, as well as for quantum technologies, offering new sources of entangled states^13,22–26,27^. The special dispersion relation in PTCs arises from interference between multiple time-reflected and time-refracted EM waves which are generated from the abrupt variations to the refractive index^20,21,28–38^. The exponentially growing modes associated with the momentum gap are possible because the time-symmetry is broken by the modulation of the refractive index. Namely, the growing modes extract energy from the index modulation, while the decaying modes transfer energy to it. Importantly, this energy exchange between the gap modes and the index modulation is non-resonant, hence it can support numerous visionary ideas such as lasers that do not rely on any atomic resonance^25^, non-resonant creation of pairs of entangled photons^25^, etc. Recently, studies of the nonlinear phenomenon of solitons in nonlinear PTCs have demonstrated the unique properties of the momentum gaps in PTCs and predicted the existence of superluminal $k$-gap solitons^39^.

The unusual dispersion relation in PTCs was thus far not utilized in the context of nonlinear frequency conversion—a core concept in nonlinear optics and in fact the first nonlinear optical phenomenon to be discovered^40^. Nonlinear frequency conversion is strongly affected by phase-matching. Generally, phase matching depends on the dispersion in the medium and often does not occur naturally. Over the years, many methods for phase-matching have been explored, the most important ones being birefringence phase-matching and quasi-phase-matching. The most basic and commonly used nonlinear frequency conversion process is second harmonic generation (SHG), where due to the non-centrosymmetric structure of certain materials, a propagating wave with frequency $\omega$ excites the spatially asymmetric dipoles in the medium, which, in turn, emits radiation at frequency $2\omega$.

Here, we explore SHG in photonic time crystals and find that SHG can be exponentially enhanced when the momentum gap modes are involved. We find the phase matching conditions for the Floquet modes associated with the momentum bands and the momentum gaps of the PTC. We show how the momentum bandgaps in PTCs can enable exponentially amplified SHG even without phase matching, extracting energy from the modulation of the refractive index. The amplification in the gaps is non-resonant, and without any threshold requirements. Moreover, we observe a dramatic cascading effect of the emergence of higher order harmonics with wavenumbers *nk*_0_, where *k*_0_ is the wavenumber of the fundamental mode (that resides within the momentum gap of the PTC), and *n* is an integer. Each harmonic is growing exponentially, with no saturation effect, drawing the energy from the modulation of the PTC. Finally, we discuss how the exponential amplification of the SHG process can pave the way towards designing momentum bandgaps at multiple wavelengths simultaneously by employing cascaded ${\chi }^{(2)}$ processes, and envision new physical mechanisms for high-harmonic generation in solids and exploiting them for ultrashort laser pulses.

## Results

For simplicity, we consider a nonlinear coefficient ${\chi }^{(2)}$ that is constant in time and does not depend on frequency, as is the case far from atomic resonances or bandgaps in solids. For clarity, we define the frequency and momentum of the original wave as $\,{\omega }_{0}$ and ${k}_{0}$, respectively, and the frequency and momentum of the generated wave as ${\omega }_{SH}$ and ${k}_{SH}$. It is important to note that the true frequency of each of the waves is well-defined only when the medium is stationary in time, because the frequency varies during the modulation.

In the conventional SHG process, we consider a finite nonlinear medium, breaking homogeneity in space (at the entrance and exit planes) but time-translation symmetry is conserved, hence the modes are defined by their frequencies. Thus, the solution to the SHG process is a time-harmonic wave with a well-defined single frequency ${\omega }_{SH}=2{\omega }_{0}$. The generated wave has a spatially varying envelope, that changes as the second harmonic (SH) mode draws energy from the fundamental mode (pump). The phase-matching condition ensures efficient transfer of energy from the fundamental mode to the SH mode, in which case its spatial envelope grows in the medium. If the phase-matching condition is not met, energy is transferred back and forth between the fundamental and SH modes, and the spatial envelope oscillates in space.

The logic in the SHG process in time-varying media is different. In a time-varying medium, it is natural to work with spatially homogeneous initial conditions with no spatial boundaries, which implies that the modes are defined by their wavenumbers. Hence, the solution for the nonlinear process is a spatially harmonic wave with a well-defined wavenumber ${k}_{SH}=2{k}_{0}$. The generated SH wave now has a time-varying envelope (in analogy to conventional SHG in stationary media). Here, phase-matching conditions will depend on the temporal frequencies of the fundamental and SH modes, as we show below for PTCs. For additional discussion on boundary conditions in time-varying media, see Supplementary Material.

**Fig. 1:**
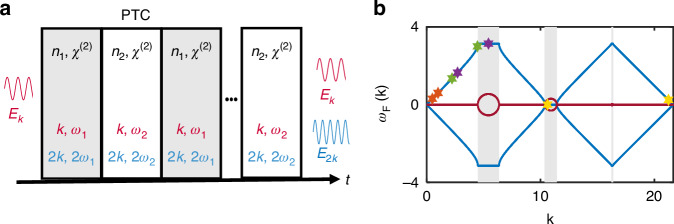
Harmonic generation in a nonlinear PTC. **a** Sketch of the system. A wave with wavenumber *k* enters a nonlinear spatially—homogeneous PTC. The nonlinearity generates a second harmonic wave, with wavenumber 2*k*. **b** Band structure of a linear PTC. In blue is the real component of the Floquet frequency ${\omega }_{F}(k)$, and in red is the imaginary component. The regions with a gray background in **b** are the momentum gaps. The stars with different colors represent different choices of momentum for the fundamental and second harmonic signal: Orange—phase-matched process between band modes. Green—phase-mismatched process between band modes. Purple—phase-mismatched process with the fundamental in a band and the second harmonic in the gap. Yellow—phase-mismatched process with the fundamental in the gap and the second harmonic in the band

For this reason, throughout this work, we consider spatially homogeneous initial conditions with no spatial boundaries. The medium is stationary until the moment $t=0$, then a PTC is established by modulating the refractive index periodically in time, $n(t)={n}_{0}+{n}_{1}(t)$, with a temporal period $T$, such that ${n}_{1}(t)={n}_{1}(t+T)$. The modulation stops after *N* cycles, at $t=NT$, such that $N$ is large (at least *N* = 20), as sketched in Fig. 1a. Due to our spatially homogeneous boundary conditions, we refer to the fundamental and SH modes by their well-defined wavenumbers: $k,2k$.

Following the modulation defining the PTC, the EM waves in a linear medium have the form of Floquet solutions (See Supplementary Material for full derivation):

1 $${E}_{k}(z,t)=u(t){e}^{i({\omega }_{F}(k)t-kz)}\,;\,{E}_{2k}(z,t)=v(t){e}^{i({\omega }_{F}(2k)t-2kz)}$$

The solutions are plane waves, multiplied by periodic functions with the periodicity of the modulation, $T$, i.e., $v(t)=v(t+T)$,$\,u(t)=u(t+T)$. The frequencies ${{\rm{\omega }}}_{F}(k)$, ${\omega }_{F}(2k)$ are the Floquet frequencies, determined from the band structure of the PTC (Fig. 1b) similar to the momentum vector of Bloch modes in spatial crystals. The Floquet frequencies differ from the true frequencies of a time-harmonic mode in a stationary medium that are derived from the static refractive index of the material. In the absence of nonlinearity, $u(t)$ and $v(t)$ are decoupled from each other.

For the nonlinear case of SHG, the electric field must satisfy the nonlinear wave equation

2 $${\nabla }^{2}\,E=\mu \left(\frac{{\partial }^{2}\varepsilon (t)}{\partial {t}^{2}}E+\varepsilon (t)\frac{{\partial }^{2}E}{\partial {t}^{2}}+2\frac{\partial \varepsilon (t)}{\partial t}\frac{\partial E}{\partial t}\right)+\mu \frac{{\partial }^{2}}{\partial {t}^{2}}({\chi }^{(2)}{E}^{2})$$

Under the non-depleted pump approximation for the fundamental mode with wavenumber $k$, the amplitude of the fundamental mode, $A$, does not depend on time, hence the solution is of the following form:

3 $${E}_{k}(z,t)=Au(t){e}^{i({\omega }_{F}(k)t-kz)}+c.c.;\,{E}_{2k}(z,t)=B(t)v(t){e}^{i({\omega }_{F}(2k)t-2kz)}+c.c.$$

Next, we substitute Eq. 3 into Eq. 2 and employ the slowly time-varying envelope approximation $|\ddot{B}|\ll |{\omega }_{F}\dot{B}|,|\dot{v}\dot{B}|$. We also keep only spatially synchronized terms containing the wavenumber $2k$. Thus, we obtain the following result, where we introduce a new function $f(t)$ and the constant $\Delta \omega$, representing the phase mismatch:

4 $$\dot{B}=-{A}^{2}{\chi }^{(2)}f(t){e}^{-i\Delta \omega t};\,f(t)\triangleq \frac{u\ddot{u}+{\dot{u}}^{2}-2{\omega }_{F}^{2}(k){u}^{2}+4i{\omega }_{F}(k)u\dot{u}}{\varepsilon (\dot{v}+i{\omega }_{F}(2k)v)+\dot{\varepsilon }v}$$

$$\Delta \omega \,\triangleq\, {\omega }_{F}(2k)-2{\omega }_{F}(k)$$

Since $f(t)$ is composed of functions with time periodicity $T$, we expand it into a Fourier series. Defining $\Omega \triangleq \frac{2{\rm{\pi }}}{{\rm{T}}}$, $f(t)=\sum _{n\in {\mathbb{Z}}}{a}_{n}{e}^{i\Omega nt}$, we obtain the amplitude of the second harmonic as a function of time *t*:

5 $$B(t)=-2i{A}^{2}{\chi }^{(2)}\sum _{n}{a}_{n}\left(\frac{{e}^{i(\Omega n-\Delta \omega )t}-1}{{\rm{i}}(\Omega n-\Delta \omega )}\right)$$

The solution for the SH field is as follows:

6 $${E}_{2k}(t)=-2i{A}^{2}{\chi }^{(2)}\sum _{n}{a}_{n}\left(\frac{{e}^{i(\Omega n-\Delta \omega )t}-1}{{\rm{i}}(\Omega n-\Delta \omega )}\right)v(t){e}^{i({\omega }_{F}(2k)t-2kz)}+c.c.$$

|$$B(t){|}^{2}$$ is related to the amount of energy transferred from the fundamental field (pump) to the SH field. In the conventional SHG derivation in stationary nonlinear media, the efficient SHG requires phase-matching $$\Delta k=2k(\omega )-k(2\omega )=0$$. In that case, the relative phase between the fundamental and the harmonic field is constant $$\frac{\pi }{2}$$, which is the optimal phase for the transfer of energy, and therefore energy is constantly transferred from the fundamental to the harmonic. In our case, if $$Re(\Delta \omega )\equiv Re({\omega }_{F}(2k)-2{\omega }_{F}(k))=n\Omega$$ for some integer $$n$$, we obtain the analogous phase matching condition in the PTC, where the oscillations $$\varepsilon (t)$$ enable this Floquet phase matching condition.

We note that phase matching in a PTC depends on the Floquet frequency, and not on the true frequency $\omega (k)$ derived from the refractive index in a stationary medium. The Floquet frequency can be controlled by changing the modulation and through the corresponding band structure of the PTC that can be engineered to phase-match a selected second harmonic (see Fig. 1b for several choices of fundamental and second harmonic momentums for a given PTC’s band structure).

More exotic cases arise when one or both momenta $k$, $2k$ are within the momentum gap of the PTC and the Floquet frequencies have an imaginary part. The linear PTC modes in the momentum gap have two branches—one with exponentially growing modes ($Im({\omega }_{F})\,>\,0$), and another with exponentially decaying modes ($Im({\omega }_{F})\,<\,0$). When we examine the form $B(t)$, we see that the envelope grows exponentially in time, if $Im(\Delta \omega )\,<\,0$, and decreases, if $Im(\Delta \omega )\,>\,0$. In those cases, even if $Re(\Delta \omega )\,\ne\,0$,i.e., we have phase mismatch, the dominance of the exponential growth overpowers any oscillations caused by the phase mismatch, and the SH process becomes efficient. Henceforth we consider four generic cases.

### Case I

**Fig. 2:**
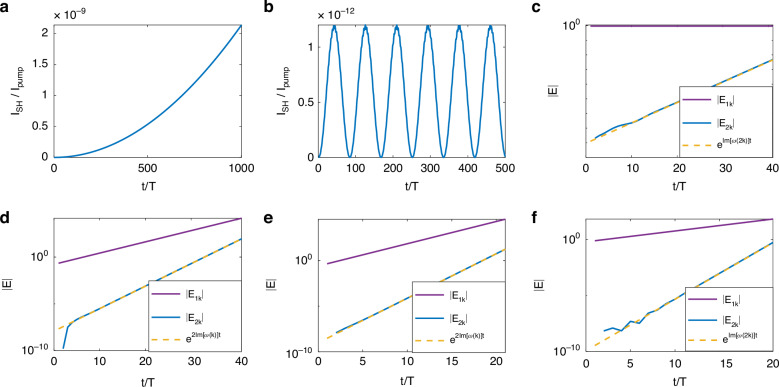
Generic cases of SHG in a PTC. **a**, **b** Case I: intensity of the SH as a function of time, with both $k$ and $2k$ in the band, with (**a**) and without (**b**) phase-matching, respectively. When the process is phase-matched (**a**) the SH intensity grows parabolically with time, whereas for a phase-mismatched process (**b**) the SH intensity oscillates in time. **c** Case II: phase-mismatched SHG with the SH in the momentum gap: The SH grows exponentially despite the phase mismatch. The vertical axis is logarithmic. **d** Case III: phase mismatched SHG with the fundamental in the gap: both the fundamental and second harmonics grow exponentially, despite the phase mismatch. The second harmonic grows with an exponential rate twice as large as the first harmonic, even though its mode belongs to the band. **e**, **f** Case IV: phase mismatched SHG with both fundamental and SH in the gap, where the dominant exponent is the exponent of the fundamental mode (**e**) and where the dominant exponent is the exponent of the SH mode (**f**)

Both the fundamental and the SH modes are within the band and their Floquet frequencies are real. In this case, the outcome is analogous to the conventional case of SHG in a stationary medium. If phase matching is fulfilled, the envelope of the SH field is linearly growing in time and the intensity is growing parabolically (Fig. 2a). If they are not phase-matched, the intensity of the SH oscillates periodically with periodicity $2\pi /\Delta \omega$, and the power is periodically transferred to and from the pump to the SH (Fig. 2b).

### Case II

The wavenumber of the fundamental mode, *k*, is within the band while the SH wavenumber $2k$ is in the momentum gap. Since the PTC’s band structure allows for modes with either negative or positive complex Floquet frequencies, this case has modes with $Im\left(\Delta \omega \, <\, 0\right.$ or $Im(\Delta \omega )\, >\, 0$. Consider first the mode with $Im(\Delta \omega )\, <\, 0$ which implies $Im({\omega }_{F}(2k))\, <\, 0$, giving rise to exponentially growing $B(t)$. In this case, despite the growth in $B(t)$ representing energy transfer from the pump to the SH, the process is also coupled to the exponentially decreasing mode in the momentum gap, and overall, this mode does not experience exponential growth and does not become a dominant mode. In the other case, where the process begins with the mode with $Im({\omega }_{F}(2k))\, >\, 0$, with decaying power transfer from the fundamental to $B(t)$, the process is coupled to the growing gap mode of the PTC. In reality, one cannot generate a case when only the decaying mode exists, because the interaction with the fundamental wave will always mix the two states. Therefore, the SH mode always has the seed it needs to exponentially grow, and the long-term dynamics are dominated by the exponentially growing mode of the SH residing in the momentum gap of the PTC (Fig. 2c).

### Case III

This case is the opposite of Case II. Here, the wavenumber $k$ of the fundamental mode is in the gap while the SH’s wavenumber $2k$ is in the band. When $Im({\omega }_{F}(k)) \,>\, 0$, $B(t)$ grows exponentially, which implies that the power transfer from the exponentially growing $k$ mode to the SH field grows exponentially, with an exponent twice the exponent of the fundamental mode (Fig. 2d).

### Case IV

This last case occurs when both the pump and the SH are in the momentum gap. In this case, the dominant exponent is either ${e}^{Im(2{\omega }_{F}(k))}$ or ${e}^{Im({\omega }_{F}(2k))}$, depending on which one is larger as shown in Eq. (6) (Fig. 2e, f).

Importantly, just like the exponential amplification of gapped modes in PTCs, the exponential amplification of the SH signal is not affected by the phase mismatch because the exponential amplification associated with gap modes overcomes the phase mismatch. This is seemingly similar to OPAs, which can also support amplification without phase-matching. However, this similarity is misleading, because in OPAs the amplification without phase-matching occurs only above a threshold (when the magnitude of the gain coefficient is larger than the phase mismatch), whereas here the amplification always occurs, without any threshold, even for large phase-mismatch. Moreover, the process here is non-resonant, and it takes place for every fundamental signal that is in the band gap.

### **Appearance of higher harmonics**

**Fig. 3:**
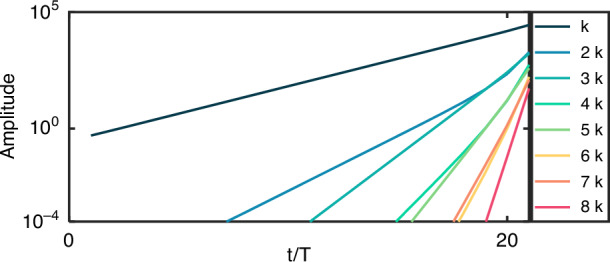
Cascaded harmonics generation: intensity of modes with different wave numbers as a function of time, sampled at the end of every PTC period. The cascading of the harmonics grows exponentially, at increasing exponential rates, with no depletion or saturation, drawing their energy from the modulation that creates the PTC

Next, we ask what happens when the second harmonic signal becomes strong due to the exponential amplification. We focus on the case where the wavenumber of the fundamental ($k$) is in the gap and the SH ($2k$) is in the band, hence the SH is growing at a faster rate than the fundamental (Case II). In the conventional SHG process, the fundamental is depleted when a non-negligible fraction of its power is transferred to the SH. However, unlike the conventional SHG, which is a parametric process and its energy is conserved and the pump is always depleted when the SH becomes stronger, here, the PTC modulation keeps driving energy into the system. Consequently, as the SH gets stronger, we observe a dominant cascading effect: higher-order harmonics emerge, and they also grow at exponential rates. For example, a signal with a wavenumber $3k$ is generated by a sum-frequency process of the fundamental and SH. Likewise, a signal with wavenumber $4k$ is generated by the sum frequency of the SH signal with itself, and so on, until we observe high-order harmonics with wavenumber $n{k}_{o}$, where $n$ is an integer. As an example, consider the generation of the 4^th^ harmonic with wavenumber $4k$, generated by cascading SHG, where the SH (2*k*) arising from SHG of the fundamental and wavenumber *k* serves as the fundamental for the new *χ*^(2)^ process. Consequentially, the fundamental signal for the new process is displaying the same exponential growth as a gapped mode. Therefore, we expect that the new nonlinear signal (with wavenumber $4k$) will grow with an exponential rate that is twice the exponent of the generating signal, i.e., with exponent ${e}^{Im(4{\omega }_{F}({k}_{0}))}$. Overall, each signal with wavenumber $n{k}_{0}$ is expected to grow at an exponential rate ${e}^{Im(n{\omega }_{F}({k}_{0}))}$, even though only the fundamental signal $k$ is in the momentum bandgap of the PTC as shown in Fig. 3. We emphasize that the power of all the different harmonics may become comparable to the power in the fundamental, yet still—the power carried by the harmonics keeps growing exponentially without any saturation, drawing energy from the modulation of the refractive index as long as the transfer of energy rate to the higher modes is lower than the rate of the growth of the fundamental harmonic.

The nonlinear cascade of harmonics exemplifies how the band structure of the PTC changes dramatically when we introduce nonlinearities. Considering for example a PTC with one bandgap around $k$, and a nonlinear coefficient ${\chi }^{(2)}\,\ne\, 0$, in the presence of an EM mode $k$, we expect now all modes with wavenumber $n{k}_{0}$ to behave as gapped modes that grow exponentially. This could have many applications. For example, it could pave the way for introducing momentum bandgaps at optical frequencies, by utilizing nonlinear ${\chi }^{(2)}$ or ${\chi }^{(3)}$ processes, which have not yet been accessed experimentally, by exploiting nonlinear ${\chi }^{(2)}$ or/and ${\chi }^{(3)}$ processes.

## Discussion

In this work, we studied the generation of a second harmonic wave in a photonic time crystal and found that the phase-matching condition depends on the Floquet frequencies in the band structure of the photonic time crystal. Recalling that the band structure of a PTC is shaped by the temporal modulation of the refractive index, this exemplifies the potential of dispersion engineering in time-varying materials, specifically in shaping nonlinear processes. Moreover, when one of the interacting waves is associated with a momentum bandgap, we observe exponential amplification of the second harmonic, followed by a cascade of higher harmonics growing at a faster exponential rate. The process does not require phase-matching or a resonance of any kind, and it does not have any threshold. In fact, in the process of nonlinear frequency conversion in PTCs, the nonlinearity acts as a mediator, facilitating energy flow from the modulation of the refractive index to the various harmonics, effectively changing the band structure. The cascading effect can pave the way for new experimental methods to achieve a momentum bandgap in the optical regime of EM waves. Moreover, the creation of an exponentially growing frequency comb in this process implies that a nonlinear PTC can be used to generate ultrashort laser pulses. Physically, the order of the harmonics generated in this cascading process can be high and limited only by the material response (via the value of ${\chi }^{(2)}$) and by the rate of the modulation of the refractive index. As such, it is possible, in principle, that this could serve as a new process for High Harmonic Generation in solids.

The concepts presented here examined SHG, but obviously, they apply to all ${\chi }^{(2)}$ processes, and higher-order processes (${\chi }^{(3)}$ and higher) can be formulated in the same way. In the broad context of nonlinear frequency conversion in time-varying media, several general intriguing questions arise. For example, thus far we considered a fixed value for the nonlinear coefficient ${\chi }^{(2)}$, but when the modulation is very strong, the atomic potential is likely to be affected, and therefore the nonlinearities will also present time-periodic dependence.

To observe the phenomena predicted here in experiments, one must first create a PTC at optical frequencies. The main challenge is that the experimental system should support exceptionally strong (on the order of unity) modulation of the refractive index within the timescale of a single optical cycle, to form a wide enough momentum band gap. Most known methods for changing the refractive index (such as the optical Kerr effect, nonlinearities in liquid crystals, or thermal nonlinearities) are either too weak or too slow. Nevertheless, the search for mechanisms that can enable fast and strong changes in the refractive index has recently been making real progress. A promising avenue in this pursuit relies on transparent conductive oxides (TCOs). In TCOs, the refractive index can be changed by order of unity at specific wavelengths, by optically exciting electrons to higher energetic states in the conduction band^1–11,17^. A recent experiment demonstrated a refractive index change of approximately 0.5 in the single-cycle time frame in TCO samples, with no material damage^8^. As mentioned, realizing a PTC requires periodic modulation, with the period of the order of a single cycle. Thus, the ultrafast change in the refractive index must be followed by ultrafast fast relaxation, and the process should be repeated at least 3–5 times to form a band structure. This requirement, in itself, creates a problem, because the relaxation process in semiconductors typically relies on interaction with phonons (a relatively slow process of ~200 femtoseconds). However, recent work has presented evidence for an ultrafast relaxation mechanism (~10 femtoseconds) in TCOs^8^ that can enable periodic modulation of the refractive index on the few-femtosecond timescale. Thus, using TCOs or similar mechanisms is a promising route to facilitate the creation of optical PTCs. Furthermore, TCOs have been shown to display strong nonlinearities^2,3,5,41,42^. From there, in order to observe the nonlinear interaction in the materials with the second-order nonlinearity, one could conduct the experiments suggested here. For centrosymmetric materials, one can focus on the dynamics of third-order nonlinear processes (third harmonic generation), which can be analyzed in a similar fashion.

The recent experimental works have also looked at nonlinear processes in TCOs, when the sample is modulated with a single modulation^43–45^. In those experiments, either second harmonic or third harmonic generation was measured in different TCO materials and in various experimental configurations. Also, these works explored different effects of the time modulation on the properties of the nonlinear processes, such as the enhancement (or suppression) of the second harmonic (or third harmonic) wave, the dependence of the harmonic intensity on the intensity of the fundamental wave, etc. The most surprising experimental results have to do with the latter. Namely, the experiments revealed that the intensity of the higher harmonic wave scales with the intensity of the fundamental wave in a totally unexpected fashion. This result presented in several studies with different materials and configurations^43–45^, is not yet understood. One thing is clear: modulating the TCO strongly affects the nonlinear frequency conversion process. This raises some additional ideas and questions: is it possible to induce large changes in nonlinear coefficients of solid materials (such as TCOs) on the single-optical-cycle timescale? Would it be possible to induce large ultrafast changes in the crystalline dipole of solids?

## Materials and methods

### Transfer-matrix method for nonlinear time-varying media

In order to simulate the SHG process in the PTC, we develop a variation of the transfer matrix method (TMM) for time-dependent media. The linear time-dependent TMM was previously used to describe a medium whose refractive index is being varied at discrete time steps. At the time-step of the change, the EM waves experience a time-interface in which part of the wave is reflected back in space, experiencing time-reflection, and part of the wave keeps propagating forward (time-refracted), but experiencing a phase shift from the time-interface.

Working with discrete time steps, we denote ${E}_{k/2k,+/-}^{n\,}$ the amplitude of the mode of wavenumber $k/2k$, propagating in the direction $+/-z$ in the n*th* time step. The electric field in the n*th* time-step is:

7 $${E}^{n}(z)=Re({E}_{k,+}^{n}{e}^{-ikz}+{E}_{k,-}^{n}{e}^{-ikz}+{E}_{2k,+}^{n}{e}^{-i2kz}+{E}_{2k,+}^{n}{e}^{-i2kz})$$

Considering EM waves with wavenumbers $k,2k$ propagating in this time-varying medium, we work with the basis:

8 $$\left(\begin{array}{c}{E}_{k,-}^{n}\\ {E}_{k,+}^{n}\\ {E}_{2k,-}^{n}\\ {E}_{2k,+}^{n}\end{array}\right)$$

At the actual time-interface, we may neglect nonlinear interactions, since the interface is instantaneous. Hence, the transfer matrix is:

9 $$T({n}_{1},{n}_{2})=\frac{1}{2}\frac{{n}_{1}}{{n}_{2}}\left(\begin{array}{cc}\begin{array}{cc}1+\frac{{n}_{1}}{{n}_{2}} & 1-\frac{{n}_{1}}{{n}_{2}}\\ 1-\frac{{n}_{1}}{{n}_{2}} & 1+\frac{{n}_{1}}{{n}_{2}}\end{array} & 0\\ 0 & \begin{array}{cc}1+\frac{{n}_{1}}{{n}_{2}} & 1-\frac{{n}_{1}}{{n}_{2}}\\ 1-\frac{{n}_{1}}{{n}_{2}} & 1+\frac{{n}_{1}}{{n}_{2}}\end{array}\end{array}\right)$$

Where ${n}_{1},{n}_{2}$ are the refractive indices before and after the time-interface The coupling between the forward and backward propagating modes results from the time-reflection and time-refraction from the time-interface.

Between two time-interfaces, the waves are propagating in a nonlinear medium with a constant refractive index. The waves accumulate phase from the propagation in the medium, and are coupled to one another via the nonlinear interaction.

For a static, homogenous, nonlinear medium, we can find the coupling between the $k,2k$ modes by solving the nonlinear wave equations, with the non-depleted pump approximation. The solution for the SH wave, assuming no material dispersion (resulting in perfect phase matching) is:

10 $${E}_{2k}(t)={e}^{i[2\omega (t-{t}_{0})-2kz]}{E}_{2k}({t}_{0})-\frac{i{\chi }^{(2)}\cdot 2\omega }{{n}^{2}}{({E}_{k}({t}_{0}))}^{2}(t-{t}_{0}){e}^{i[2\omega (t-{t}_{0})-2kz]}$$

$$\omega\, \triangleq\, \frac{ck}{n}$$

*χ*^(2)^ is the nonlinear susceptibility in the medium, and $n$ is the refractive index in the medium. ${t}_{0}$ is some fixed time at which the electric field is given.

Assuming the fundamental mode $k$ only accumulates phase, the $2k$ mode is coupled to the $k$ mode propagating in the same direction.

Thus, between two time-interfaces, we propagate the SH modes in the nonlinear manner:

11 $${E}_{k,\!\!\pm }^{i}={E}_{k,\!\!\pm }^{i-1}{e}^{\pm\!\! i\omega \Delta t}$$

12 $${E}_{2k,\!\!\pm }^{i}={E}_{2k,\!\!\pm }^{i-1}{e}^{\pm i2\omega \Delta t}-\frac{i{\chi }^{(2)}2\omega }{{n}^{2}}{({E}_{k,\!\!\pm }^{i-1})}^{2}\Delta t\,{e}^{\!\!\pm i2\omega \Delta t}$$

When $\Delta t$ is the length of the time step.

The complete algorithm, given ${n}_{i}$, the modulated refractive index sampled at discrete time steps of length $\Delta t$, goes as follows:

For every time step $i$, updating the electrical field vector defined in Eq. 8:

1. Nonlinear propagation according to Eqs. 11 and 12.
2. Updating the vector after the time-interface using the transfer matrix defined in Eq. 9.

Overall, the complete propagation from timestep $i$ to timestep $i+1$ is:

13 $$\left(\begin{array}{c}{E}_{k,-}^{i+1}\\ {E}_{k,+}^{i}\\ {E}_{2k,-}^{i}\\ {E}_{2k,+}^{i}\end{array}\right)=T({n}_{i},{n}_{i+1})\left(\begin{array}{c}{E}_{k,-}^{i}{e}^{-i\omega \Delta t}\\ {E}_{k,+}^{i}{e}^{i\omega \Delta t}\\ {E}_{2k,-}^{i}{e}^{-i2\omega \Delta t}-\frac{i{\chi }^{(2)}2\omega }{{n}_{i}^{2}}{({E}_{k,-}^{i})}^{2}\Delta t\,{e}^{-i2\omega \Delta t}\\ {E}_{2k,+}^{i}{e}^{i2\omega \Delta t}-\frac{i{\chi }^{(2)}2\omega }{{n}_{i}^{2}}{({E}_{k,+}^{i})}^{2}\Delta t\,{e}^{i2\omega \Delta t}\end{array}\right)\,;\,\omega\, \triangleq\, \frac{ck}{{n}_{i}}$$

The TMM is an efficient tool that can be extended to study other frequency-generation processes in PTCs, such as third harmonic generation. However, using the TMM approach, the user must decide in advance what will be the modes in the system (what will be their wavenumbers), and the method is efficient for the small number of modes. Therefore, to simulate wave packets, or cascading effects that include many different spatial modes, we turn to the finite-difference time-domain (FDTD) method, which we discuss in the next section.

### FDTD simulations of nonlinear, time-varying media

Before proceeding to the FDTD simulations, we note that both the analytic part (described in the Results section of the main text) and the calculations based on the transfer matrix method (described in the “Transfer-matrix method for nonlinear time-varying media” section of the “Methods”), use the non-depleted pump approximation, which is standard in nonlinear optics. However, in the current section—where we simulate the nonlinear frequency conversion process using FDTD, we solve the full coupled equations, namely, we also account for the depletion of the pump (the wave at the fundamental frequency). The results of the simulation are presented in Fig. 3 in the main text and discussed there.

For simulating the cascading effect, we develop a new the FDTD method. Consider EM waves propagating in the ±*z* direction, with their electric field in the *x* direction and magnetic field in the *y* direction: $\overrightarrow{E}={E}_{x}\hat{x}$, $\overrightarrow{H}={H}_{y}\hat{y}$. Working with the staggered grid method, for our sampled field, the notation is:

14 $${E}_{x}(t=n\cdot \Delta t,z=k\cdot \Delta z)\,\triangleq\, {E}_{n,k}$$

15 $${H}_{y}(t=\left(n+\frac{1}{2}\right)\cdot \Delta t,z=\left(k+\frac{1}{2}\right)\cdot \Delta z)\,\triangleq\, {H}_{n+\frac{1}{2},k+\frac{1}{2}}$$

From Maxwell equations:

16 $$\frac{\partial {E}_{x}}{\partial z}=-\mu \frac{\partial {H}_{y}}{\partial t}$$

17 $$\frac{\partial {H}_{y}}{\partial z}=-\frac{\partial }{\partial t}D(t)$$

And the relation:

18 $$D(t)={\varepsilon }_{L}E(t)+{\chi }^{(2)}{E}^{2}$$

We update the electric and magnetic fields in the following manner:

19 $${H}_{n+\frac{1}{2},k+\frac{1}{2}}={H}_{n-\frac{1}{2},k+\frac{1}{2}}-\frac{\Delta t}{\Delta z}\cdot \frac{1}{{\mu }_{0}}({E}_{n,k+1}-{E}_{n,k})$$

20 $${E}_{n+1,k}={E}_{n,k}-\frac{\Delta t}{\Delta z}\cdot \frac{1}{{\varepsilon }_{n,k}}({H}_{n+\frac{1}{2},k+\frac{1}{2}}-{H}_{n+\frac{1}{2},k-\frac{1}{2}})$$

To simulate a time-varying and nonlinear medium, we use ${\varepsilon }_{n,k}$ that varies in time and space:

21 $${\varepsilon }_{n,k}={\varepsilon }_{n}^{linear}+{\chi }^{(2)}{E}_{n,k}$$

The first term, ${\varepsilon }_{n}^{linear}$, is a time-dependent function, for example ${\varepsilon }_{0}+\,\cos (\Omega t)$, representing the PTC modulation of the linear refractive index. The second term, ${\chi }^{(2)}{E}_{n,k}$ represents the nonlinearity of the susceptibility, which is affected by the electrical field.

## Supplementary information

Supplemental Material

## Data Availability

Simulation data is available upon request.

## Code availability

Details of the code are given in the “Methods” section.
