# Supplementary material for: Second harmonic generation and nonlinear frequency conversion in photonic time-crystals
Source: Light Sci Appl. 2025 Apr 2;14:152. doi: 10.1038/s41377-025-01788-z (PMC11965301; doi:10.1038/s41377-025-01788-z)
Supplement: Supplementary file 1 — Supplemental Material [file 41377_2025_1788_MOESM1_ESM.docx]

**Second Harmonic Generation and Nonlinear Frequency Conversion in Photonic Time-Crystals**

**Noa Konforty^1,2^, Moshe-Ishay Cohen^1,2^, Ohad Segal^2,3^, Yonatan Plotnik^2^, Vladimir M. Shalaev^4,5^ and Mordechai Segev^1,2,3^**

^1^ Physics Department, Technion – Israel Institute of Technology, Haifa 32000, Israel

^2^ Solid State Institute, Technion – Israel Institute of Technology, Haifa 32000, Israel

^3^ Department of Electrical and Computer Engineering, Technion – Israel Institute of Technology, Haifa 32000, Israel

^4^ School of Electrical and Computer Engineering, Birck Nanotechnology Center and Purdue Quantum Science and Engineering Institute, Purdue University, West Lafayette, IN 47907, USA

^5^ Quantum Science Center (QSC), a National Quantum Information Science Research Center of the U.S. Department of Energy (DOE), Oak Ridge, TN 37931, USA

**Supplementary Information**

In this supplementary information, we provide additional details regarding the theoretical derivations in the main text.

1. **Boundary Conditions in Nonlinear Media**

In the conventional second harmonic generation (SHG) process, we consider a finite nonlinear medium, breaking homogeneity in space (at the entrance and exit planes) but time-translation symmetry is conserved, hence the conserved quantity is frequency (or energy). In such setting, we consider an electromagnetic (EM) wave propagating into the medium, and exiting after propagating for a finite distance.

Thus, the solution to the SHG process is a time-harmonic wave with a well-defined single frequency $\omega_{f}=2\omega_{i}$, where $\omega_{i},\omega_{f}$ are the frequencies of the fundamental (pump) and generated waves, respectively. The generated wave has a spatially-varying envelope, that varies with propagation as the second harmonic (SH) mode draws energy from the fundamental mode (pump). The form of the SH wave is therefore:

$$E_{2\omega}\left( z,t \right)=A\left( z \right)e^{i\left( 2\omega t-k\left( 2\omega\right)z \right)}+c.c. \left( S1 \right)$$

Deriving $A(z)$, the spatially varying envelope for the SHG process, one finds that its shape varies according to the phase-matching condition:

$$\Delta k=k_{f}-2k_{i}=k\left( 2\omega_{i} \right)-2k\left( \omega_{i} \right)=0 \left( S2 \right)$$

$k_{i},k_{f}$ are the fundamental (pump) and SH’s wavenumbers.

The phase-matching condition ensures efficient transfer of energy from the fundamental mode to the SH mode, in which case its spatial envelope grows with the propagation distance in the medium. If the medium is dispersive, the phase matching condition is not met, energy is transferred back and forth between the fundamental and SH modes, and the spatial envelope oscillates throughout propagation. On a microscopic scale, the phase matching conditions dictates whether the radiation from the nonlinear dipoles in the medium interfere constructively, or distractively.

When considering a time-varying medium, it is easiest to work with plane-waves, hence we assume an infinite spatially-homogeneous medium in all three dimensions, where the wave-momentum is conserved. Thus, we need only to define an initial condition in time, and solve for the temporal evolution. With these boundary conditions and homogeneity in space, the solution for the nonlinear process in a single spatial dimension is a spatially-harmonic wave with a well-defined wavenumber $k_{f}=2k_{i}$. However, because time-translation symmetry is broken by the temporal changes in the permittivity, the generated SH wave now has a time-varying envelope (in analogy to conventional SHG in stationary media).

$$E_{2k_{i}}\left( z,t \right)=A\left( t \right)e^{i\left( \omega\left( 2k_{i} \right)t-2k_{i}z \right)}+c.c. \left( S3 \right)$$

In the time-varying system, the phase-matching condition depends on the temporal frequencies of the fundamental and SH modes, and has to be found for different time-dependent systems individually. In this work, we find the phase matching condition for the periodic case of a photonic time crystal (PTC).

Once the phase matching condition is satisfied, the envelope’s solution will be a growing envelope in time. If the phase matching condition is not met and the system lacks momentum gaps (as is the case, e.g., with a stationary system), the envelope oscillates in time. However, as we see in the main text, the presence of momentum band gaps can create exponentially growing modes even for the phase mismatch case.


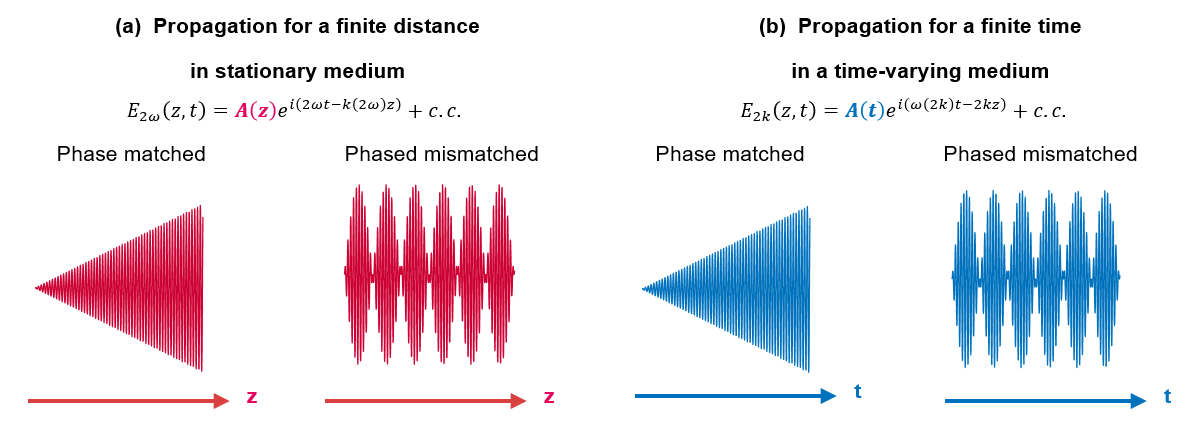


**Figure S1**: (a) Spatially-varying envelope of the emerging SH wave after a finite distance in a nonlinear stationary medium. When phase-matched, the field envelope grows linearly (under the non-depleted pump approximation) with propagation distance $\left( z \right)$, whereas when phase mismatched, the envelope oscillates. (b) Time-varying envelope in the SH wave emerging in a homogeneous medium. When phase-matched, the envelope grows as a function of time $\left( t \right)$, whereas when phase-mismatched the envelope oscillates

1. **Derivation of SH field in PTC, under the non-depleted-pump approximation**

We consider a PTC in a homogeneous medium with a time-dependent index of refraction $n\left( k,t \right)=n\left( k,t+T \right)$. $T$ is the period of the PTC, and we define the frequency of the PTC as $\Omega=\frac{2\pi}{T}$. The material is far from any resonance, and thus it may also have dispersion that can be dependent on the momentum $k$, which is a function of the instantaneous frequencies of the EM field $\omega$. The material is also non-linear, but far from any atomic resonance or bandgap, we have a non-linear $\chi^{\left( 2 \right)}$ term that does not depend on the frequency. We assume the material has no losses.

First, we look at the PTC EM modes with a linear material (without $\chi^{(2)}$). In this case the EM modes are Floquet modes. They satisfy the linear wave equation $\nabla^{2}\mathbf{E}=\mu\frac{\partial^{2}}{\partial t^{2}}\left( \varepsilon\left( t \right)E\left( t \right) \right)$, and yield the linear PTC band structure (see for example Fig 1B in the main text). We denote $E_{k}$ as the EM mode with wave number $k$, and $v\left( t \right),u\left( t \right)$ are periodic functions, with the periodicity of the PTC, i.e., $v\left( t \right)=v\left( t+T \right),u\left( t \right)=u\left( t+T \right)$. $\omega_{F}\left( k \right),\omega_{F}\left( 2k \right)$ are the Floquet frequencies, determined from the band structure of the PTC. The complete Floquet solution, including the functions $v\left( t \right),u(t)$ are found by solving the wave equation for the linear PTC.

$$E_{k}\left( z,t \right)=u\left( t \right)e^{i\left( \omega_{F}\left( k \right)t-kz \right)}+c.c. ; E_{2k}\left( z,t \right)=v\left( t \right)e^{i\left( \omega_{F}\left( 2k \right)t-2kz \right)}+c.c. \left( S4 \right)$$

Next, we look at the full nonlinear wave equation of our nonlinear PTC (with $\chi^{(2)}$).

$$\nabla^{2} E=\mu\left( \frac{\partial^{2}\varepsilon\left( t \right)}{\partial t^{2}}E+\varepsilon\left( t \right)\frac{\partial^{2}E}{\partial t^{2}}+2\frac{\partial\varepsilon\left( t \right)}{\partial t}\frac{\partial E}{\partial t} \right)+\mu\frac{\partial^{2}}{\partial t^{2}}\left( \chi^{\left( 2 \right)}E^{2} \right) \left( S5 \right)$$

We use the following ansatz, assuming $B\left( t \right)$ is some slow-varying envelope. Working under the non-depleted-pump (NDP) approximation, $E_{k}$ (the pump) is unaffected by the nonlinear process and therefore has no additional temporal envelope.

$$E_{k}\left( z,t \right)=Au\left( t \right)e^{i\left( \omega_{F}\left( k \right)t-kz \right)}+c.c. ; E_{2k}\left( z,t \right)=B\left( t \right)v\left( t \right)e^{i\left( \omega_{F}\left( 2k \right)t-2kz \right)}+c.c. \left( S6 \right)$$

When substituting those into the wave equation, we look only for spatially synchronous terms. Since we are interested in the SH field, we only look at terms that contain $e^{i2kz}$. Physically, this is because only spatially synchronous terms have constructive interference between all the homogenously-distributed dipoles induced by the fundamental wave in the medium.

$B\nabla^{2}\left( ve^{i\left( \omega_{F}\left( 2k \right)t-2kz \right)} \right)= B\mu\ddot{\varepsilon}ve^{i\left( \omega_{F}\left( 2k \right)t-2kz \right)}+\mu\varepsilon B\partial_{t}^{2}\left( ve^{i\left( \omega_{F}\left( 2k \right)t-2kz \right)} \right)+\frac{B2\mu\dot{\varepsilon}\partial}{\partial t}\left( ve^{i\left( \omega_{F}\left( 2k \right)t-2kz \right)} \right)+\mu\varepsilon\left( 2\dot{B}\partial_{t}\left( ve^{i\left( \omega_{F}\left( 2k \right)t-2kz \right)} \right)+\ddot{B}ve^{i\left( \omega_{F}\left( 2k \right)t-2kz \right)} \right)+2\mu\dot{\varepsilon}\dot{B}ve^{i\left( \omega_{F}\left( 2k \right)t-2kz \right)}+\mu\partial_{t}^{2}\left( \chi^{\left( 2 \right)}A^{2}u^{2}e^{i\left( 2\omega_{F}\left( k \right)t-2kz \right)} \right) \left( S7 \right)$

Since $v\left( t \right)e^{i\left( \omega_{F}\left( 2k \right)t-2kz \right)}$ is a solution to the linear wave equation, we can remove the first four terms, and rewrite the equation:

$$\varepsilon\left( 2\dot{B}\partial_{t}\left( ve^{i\omega_{F}\left( 2k \right)t} \right)+\ddot{B}ve^{i\omega_{F}\left( 2k \right)t} \right)+2\dot{\varepsilon}\dot{B}ve^{i\omega_{F}\left( 2k \right)t}+A^{2}\partial_{t}^{2}\left( \chi^{\left( 2 \right)}u^{2}e^{i2\omega_{F}\left( k \right)t} \right)=0 \left( S8 \right)$$

From the slowly-varying amplitude assumption, $|\ddot{B}|\ll{|\omega}_{F}\partial_{t}B| , \dot{|v}\partial_{t}B|$, and we are left with:

$$\dot{B}=-A^{2}\chi^{\left( 2 \right)}e^{i\left( 2\omega_{F}\left( k \right)-\omega_{F}\left( 2k \right) \right)t}\frac{-2\omega_{F}^{2}\left( k \right)u^{2}+i{4\omega}_{F}\left( k \right)u\dot{u}+u\ddot{u}+\dot{u}^{2}}{\dot{\varepsilon}v+\varepsilon\left( \dot{v}+i\omega_{F}\left( 2k \right)v \right)} . \left( S9 \right)$$

Notice that $\varepsilon,u,v$ are periodic in time ($u,v$ are known from the solutions for the linear PTC), and so are their derivatives, we can construct a function $f\left( t \right)$ from the expression on the right-hand side, and decompose it into a Fourier series

$$f\left( t \right)=\frac{-2\omega_{F}^{2}\left( k \right)u^{2}+i{4\omega}_{F}\left( k \right)u\dot{u}+u\ddot{u}+\dot{u}^{2}}{\dot{\varepsilon}v+\varepsilon\left( \dot{v}+i\omega_{F}\left( 2k \right)v \right)} =\sum_{n\mathbb{\in Z}} a_{n}e^{i\Omega nt} \left( S10 \right)$$

By solving the set of ordinary differential equations, we find the solution for $B(t)$.

$$B=A^{2}\chi^{\left( 2 \right)}\sum_{n\mathbb{\in Z}} a_{n}e^{\frac{1}{2}i\left( 2\omega_{F}\left( k \right)-\omega_{F}\left( 2k \right)+n\Omega\right)t}\mathrm{sinc} \left( -\frac{1}{2}i\left( 2\omega_{F}\left( k \right)-\omega_{F}\left( 2k \right)+n\Omega\right)t \right) \left( S11 \right)$$
